# Activity Analysis and Inhibition Mechanism of Four Novel Angiotensin I-Converting Enzyme Inhibitory Peptides Prepared from *Flammulina velutipes* by Enzymatic Hydrolysis

**DOI:** 10.3390/foods14152619

**Published:** 2025-07-26

**Authors:** Yajie Zhang, Xueqi Zhao, Xia Ma, Jiaqi Li, Xiaoyu Ye, Xuerui Wang, Wenwei Zhang, Jianmin Yun

**Affiliations:** College of Food Science and Engineering, Gansu Agricultural University, Lanzhou 730070, China; zhangyajie1302@163.com (Y.Z.); zhaoxqsjz@163.com (X.Z.); 19171737331@163.com (X.M.); 15709302274@163.com (J.L.); 15012368470@163.com (X.Y.); 18418651286@163.com (X.W.); zhangww@gsau.edu.cn (W.Z.)

**Keywords:** *Flammulina velutipes*, ACE inhibitory peptide, enzymatic hydrolysis, structural characterization, molecular docking, synthesis validation

## Abstract

In order to innovatively develop high-activity ACE inhibitory peptides from edible fungi, the conditions for a double-enzymatic hydrolysis preparation of ACE inhibitory peptides from *Flammulina velutipes* were optimized by response surface methodology. After purification by macroporous resin, gel chromatography, and RP-HPLC, a crude peptide fraction was obtained; its ACE inhibition rate was 85.73 ± 0.95% (IC_50_ = 0.83 ± 0.09 mg/mL). Based on LC-MS/MS sequencing, the four novel peptides, namely, FAGGP, FDGY, FHPGY, and WADP, were screened by computer analysis and molecular docking technology. The four peptides exhibited a binding energy between −9.4 and −10.3 kcal/mol, and formed hydrogen bonds with Tyr523, Ala354, and Glu384 in the S1 pocket, Tyr520 and His353 in the S2 pocket, and His383 in the HEXXH zinc-coordinating motif of ACE, indicating their good affinity with the ACE active site. The IC_50_ values of the four ACE inhibitory peptides were 29.17, 91.55, 14.79, and 41.27 μM, respectively, suggesting that these peptides could potentially contribute to the development of new antihypertensive products.

## 1. Introduction

Bioactive peptides have many advantages, including a broad array of varieties, extensive application prospects, high efficiency, non-toxicity, and good stability [1,2]. These peptides are believed to possess significant potential for their use in regulating various physiological and metabolic functions of the human body [3].

Hypertension is a chronic disease that is prevalent in the middle-aged and elderly population and seriously endangers human health [4,5,6,7]. Angiotensin I-converting enzyme (ACE) plays a pivotal role in regulating human blood pressure, and serves as an ideal target for the treatment of hypertension [8]. ACE can produce potent vasoconstrictor angiotensin II by catalyzing angiotensin I, resulting in increased blood pressure. Inhibition of ACE can reduce angiotensin II production [9,10]. Angiotensin I-converting enzyme inhibitory (ACEI) peptides have attracted increased research attention owing to their ability to effectively inhibit ACE activity and their significant effects on the prevention and treatment of hypertension [11]. ACEI can effectively inhibit the activity of ACE. At present, synthetic ACEI is mainly used to treat hypertension. However, when compared with synthetic drugs such as captopril, etc., food-derived natural ACE inhibitory peptides can minimize many side effects, including dizziness and kidney damage [12]. Nowadays, antihypertensive peptides derived from natural foods have received more and more attention due to their advantages of safety and easy availability [13,14]. Therefore, it is particularly urgent to find new and safe natural new active compounds.

To date, a variety of antihypertensive peptides have been obtained from different sources, including plants (e.g., sesame [15] and quinoa [16]), dairy products (e.g., cheese [17] and peanut yogurt [18]), marine organisms (e.g., grouper [19]) and squid [20]), animals (e.g., beef [21] and small-aroma chicken [22]), and microorganisms (e.g., *Agaricus bisporus* [23]) and *Lentinula edodes* [24]). Edible fungi are rich in proteins. After catalytic degradation by proteases, these proteins can become precursors of some functional peptides, which usually contain a large number of hydrophobic amino acids [25,26]. The peptides derived from edible fungi have been found to present numerous beneficial characteristics, including immunomodulatory, antibacterial, and hormonal regulatory properties [27]. *Flammulina velutipes*, commonly known as enoki mushroom, is the third most popular edible fungus species in the world. It is rich in nutrients such as carbohydrates, proteins, amino acids, and vitamins, and has significant medicinal and edible characteristics [28]. It also contains health-beneficial bioactive compounds, including anti-tumor, anti-oxidation, and anti-aging effects [29]. The protein components of *F. velutipes* can be used to produce a variety of ACE inhibitory peptides via enzymatic hydrolysis, making them an ideal raw material for the production of antihypertensive peptides with broad development prospects [30].

However, although there have been many studies on ACE inhibitory peptides, there is a lack of research on ACE inhibitory peptides from edible fungi, especially from *F. velutipes*. The purpose of this study was to obtain ACE inhibitory peptides with high activity from *F. velutipes* by enzymatic hydrolysis, and to identify the sequence of the purified products. Then, the ACE inhibitory activity of putative ACEI peptides was evaluated with synthetic peptides, and their ACE inhibitory mechanism was elucidated based on molecular docking technology. The findings of this study not only provide a theoretical basis and reference for the development of new antihypertensive functional foods and drugs, but also offer a novel approach for the high-value utilization of *F. velutipes*.

## 2. Materials and Methods

### 2.1. Materials and Reagents

The artificially cultivated fruiting bodies of *F. velutipes* were collected from Gansu Tianshui Zhong Xing Fungi Technology Co., Ltd. (34.58° N, 105.74° E, Tianshui, China). ACE, complex protease (120 U/mg), papain (800 U/mg), neutral protease (100 U/mg), pepsin (30,000 U/g), trypsin (4000 U/g), alkaline protease (200 U/mg), Flavor^TM^ protease (15,000 U/g), and N-[3-(2-furylacryloyl)]-L-phenylalanyl-glycyl-glycine (FAPGG) were procured from Shanghai Yuanye Biotechnology Co., Ltd. (Shanghai, China).

### 2.2. Extraction of F. velutipes Protein

**Figure 1:**
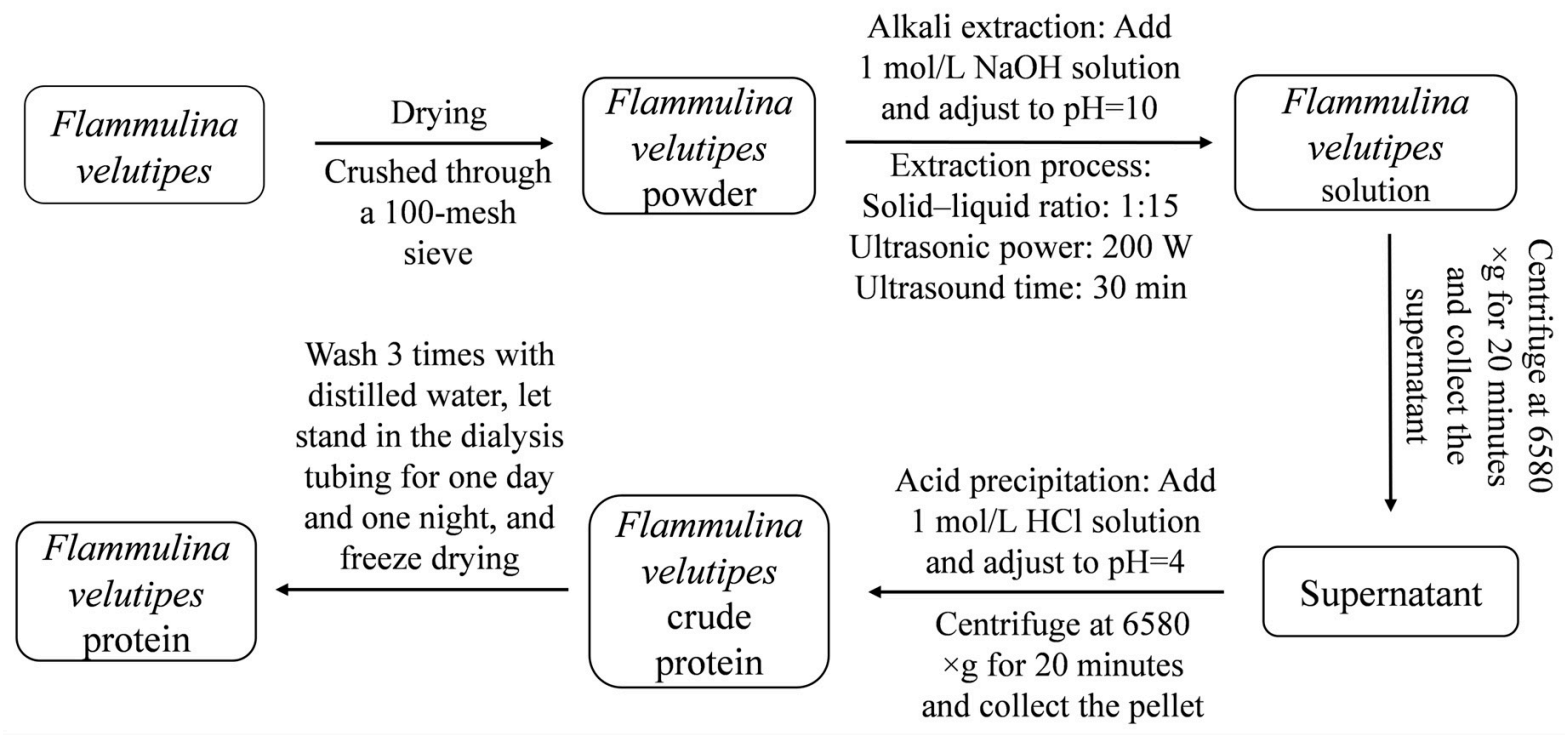
Process flow diagram of protein extraction from *F. velutipes*.

As shown in Figure 1, *F. velutipes* protein was extracted by ultrasonic-assisted alkali extraction and the acid precipitation method.

### 2.3. Screening of Proteases

The extracted *F. velutipes* protein was converted into a powder form by freeze-drying, and the substrate concentration was calibrated to a mass fraction of 2%. A total of seven proteases (pepsin, trypsin, flavor protease, papain, alkaline protease, neutral protease, and complex protease) were selected, the amount of enzyme added was 4000 U/g, and the reaction system was adjusted to the optimal pH value and enzymolysis temperature for each protease. The *F. velutipes* protein was hydrolyzed for 3 h, after which the enzyme was inactivated for 10 min at 95 °C. Subsequently, the crude hydrolysate was cooled and centrifuged (the model of the centrifuge is TGL-16M, Hunan Xiangyi Laboratory Instrument Development Co., Ltd., Changsha, China) at 1700× *g* for 20 min, the supernatant after centrifugation was used as the hydrolysate of *F. velutipes*, and then the degree of hydrolysis (DH) and ACE inhibition rate of *F. velutipes* protein were ascertained, and the optimal protease was selected.

### 2.4. Determination of Degree of Hydrolysis of Protein in F. velutipes

The DH was determined by formaldehyde titration and calculated as follows [31]:

(1) DH(%)=A/B×100

where *A* represents the content of amino nitrogen in the supernatant of enzymatic hydrolysis and *B* represents the total nitrogen content in the sample.

### 2.5. Determination of ACE Inhibitory Peptide Activity

The ACE inhibition rate was determined according to the method developed by Wang [32]. In brief, 1 mmol/L FAPGG solution, 0.1 U/mL ACE solution, and *F. velutipes* protein solution were mixed in 80 mmol/L HEPES buffer (pH 8.2), and the mixture was added to 96-well plates according to Table 1. The absorbance value at the wavelength of 340 nm at 37 °C and the absorbance value after 30 min of reaction were measured. The calculation formula is as follows:

(2) $$Inhibition\;Rate\;of\;ACE\left(\%\right)=(1-\frac{\Delta A}{\Delta B})\times 100$$

In the formula, Δ*A* = *A*_1_ − *A*_2_; Δ*B* = *B*_1_ − *B*_2._ Δ*A* was the absorbance value of the sample pore solution; Δ*B* was the absorbance value of the control pore solution. *A*_1_ and *B*_1_ were the absorbance values measured at the wavelength of 340 nm. *A*_2_ and *B*_2_ were the absorbance values measured at the wavelength of 340 nm after reacting for 30 min at 37 °C.

**Table 1:** Determination of ACE inhibition rate.

Reagent	*A* Sample (μL)	*B* Control (μL)
ACE (0.1 U/mL)	10	10
FAPGG (1 mmol/L)	50	50
ACEI	40	0
HEPES buffer (80 mmol/L)	0	40

ACEI: *F. velutipes* ACE inhibitory peptides; *A*: sample group; *B*: control group. The IC_50_ value indicated the mass concentration of the *F. velutipes* protein at which 50% ACE inhibition was achieved.

### 2.6. Response Surface Optimization

Preliminary research yielded three factors, namely, the enzymolysis time (*A*), enzymolysis temperature (*B*), and enzymolysis pH (*C*), which were selected for the optimization of the process parameters for the production of ACE inhibitory peptides using dual-enzymatic hydrolysis. The optimization was based on response surface design, and the ACE inhibition rate was used as the evaluation index. The coding values of the experimental factor levels are shown in Table 2.

### 2.7. Purification of ACE Inhibitory Peptides Derived from F. velutipes

#### 2.7.1. Macroporous Resin Separation

Five different resins (NKA-9, DA 201-C, D101, AB-8, and X-5; Shanghai Yuanye Biotechnology Co., Ltd., Shanghai, China) were used for static adsorption experiments. The adsorption rate was calculated using the following formula to determine the best resin:

(3) Adsorption rate (%) = *A* − *B*/*A* × 100

where *A* represents the protein concentration in the enzymatic hydrolysate; *B* indicates the protein concentration in the adsorption solution.

The selected resin wet packing column (2.6 × 30 cm, Beijing Ruida Henghui Technology Development Co., Ltd., Shanghai, China) was used to purify the *F. velutipes* polypeptide (30 mg/mL) under the following conditions: 0.5 BV (Bed Volume) injection volume, 1 BV/h injection flow rate, 2 mL/min eluent flow rate, and the eluent was 75% ethanol. The obtained polypeptide solution was subjected to rotary evaporation to remove ethanol and stored for later use after concentration.

#### 2.7.2. Gel Chromatography Purification and Classification

To further purify the components after resin separation, Sephadex G-100 was used to grade the separated polypeptide solution (50 mg/mL). The polypeptide solution was filtered through a 0.45 μm filter (Solarbio Science & Technology Co., Ltd., Beijing, China), with distilled water serving as the eluent. The eluent flow rate was controlled at 1 mL/min, and 3 mL of the solution was collected in each tube, and 60 tubes were collected. The three elution peak components that appeared at a wavelength of 280 nm were collected and labeled as F1 (tube 19), F2 (tube 31), and F3 (tube 42), respectively. All the collected components were sampled to determine their in vitro ACE inhibition rate, and the components with the highest activity were selected, to collect the most active components, and stored after freeze-drying.

#### 2.7.3. Purification by RP-HPLC

The F3 fraction with the highest activity after purification and classification by gel chromatography was separated by RP-HPLC, and two peaks a1 and a2 were collected to determine the ACE inhibitory activity and IC_50_ value. Chromatographic column: Amethyst C18-H, 4.6 × 250 mm, 5 μm, 120 Å; mobile phase: A: 2% acetonitrile + 98% water + 0.05% TFA; b: 90% acetonitrile + 10% water + 0.05% TFA. The elution procedure was as follows: 0–4 min: 10% B; 30 min: 70% B; 35–40 min: 90% B; 41–46 min: 10% B. Detection wavelength: UV 214 nm; flow rate: 0.8 mL/min; sampling concentration: 2 mg/mL; injection volume: 10 μL; column temperature: 25 °C.

### 2.8. Determination of Molecular Weight of Polypeptide Before and After Purification

The molecular weight of the polypeptide before and after purification was determined by gel filtration–HPLC with a calibrated column and entrusted to Beijing Biotech Pack Biotechnology Co., Ltd., (Beijing, China). An appropriate amount of sample was weighed and dissolved in water to 2 mg/mL. Liquid chromatography conditions: column temperature: 25 °C; flow rate: 0.7 mL/min; injection volume: 10 μL; chromatographic column: SRT-C SEC-120, 5 μm, 7.8 × 300 mm; mobile phase: 150 mmol/L phosphate mobile phase (8.99 g anhydrous sodium dihydrogen phosphate and 10.65 g anhydrous sodium dihydrogen phosphate to 1 L water); detection wavelength: UV 214 nm.

### 2.9. De Novo Peptide Sequencing

The a1 peak detected by RP-HPLC was selected for sequencing. The sample was dissolved in ultrapure water to 2 mg/mL, DTT solution was added, reduced in a water bath at 56 °C for 1 h, and alkylated with 50 mM IAM (iodoacetamide) at dark room temperature for 40 min. After desalination with a C18 stage tip, the sample was vacuum-dried at 45 °C, and then the sample was redissolved in 0.1% formic acid and analyzed by LC-MS/MS. The mass spectrometer used was Easy-nLC 1200/QExactive (Thermo Fisher Scientific, Waltham, MA, USA). Beijing Biotech Pack Biotechnology Co., Ltd. (Beijing, China) was entrusted for sequencing.

### 2.10. Identification of ACE Inhibitory Peptides with High Activity Using in Silico Analysis

Firstly, the Peptide Ranker (http://distilldeep.ucd.ie/PeptideRanker/, accessed on 18 March 2025) was used to identify the peptide sequence and predict its activity, with a higher score indicating higher peptide activity. A peptide score ≥ 0.8 was considered to exhibit biological activity [33]. Then, the sequences of these peptides were compared with three databases, namely PepBank (https://pepbank.mgh.harvard.edu/, accessed on 18 March 2025), Peptide DB (https://www.peptidedb.com/, accessed on 18 March 2025), and BIOPEP (https://www.uwm.edu.pl/biochemia/index.php/en/biopep, accessed on 18 March 2025) to screen new peptide sequences.

### 2.11. Molecular Docking Technology for ACE Inhibitory Peptides from F. velutipes

To further study the interaction mechanism between the prepared *F. velutipes* ACE inhibitory peptides and the ACE active site, the 3D structure of ACE (PDB ID: 1O8A) was downloaded from PDB (https://www.rcsb.org/, accessed on 19 March 2025). Before docking, the 3D structure of the ligand was constructed using ChemDraw 23.1.1 software, and MM2 was employed to minimize the energy. AutoDock Tools 1.5.7 was used to hydrogenate the ligand, modify the atom, and calculate the charge. The coordinates of the docking center were set as x = 40.589, y = 37.374, and z = 43.448, and AutoDockVina was used for docking and the binding energy was calculated. PyMol 1.5 was employed to draw a 3D map of the interaction between the peptides and ACE, and Discovery Studio 2019 was utilized to construct a 2D map.

### 2.12. Synthesis and Activity Verification of ACE Inhibitory Peptides Derived from F. velutipes

Wuhan Dangang Biotechnology Co., Ltd. (Wuhan, China) was commissioned to synthesize the peptides. The purity of the synthesized peptides was more than 95% by using the solid-phase synthesis method. With captopril as the positive control, the ACE inhibition rate and IC_50_ of the above synthetic peptides were determined according to the method of Section 2.5.

### 2.13. Statistical Analysis

Each experiment was repeated thrice to eliminate the effect of random errors, and the results are expressed as mean ± SD. One-way analysis of variance with Duncan’s multiple comparison test was performed on the data using IBM SPSS Statistics 27, and the difference was considered significant if *p* < 0.05. All the data were processed using Origin 2021 software, and the IC_50_ values were calculated utilizing GraphPad Prism 10.1.2.

## 3. Results

### 3.1. Screening of Proteases

**Figure 2:**
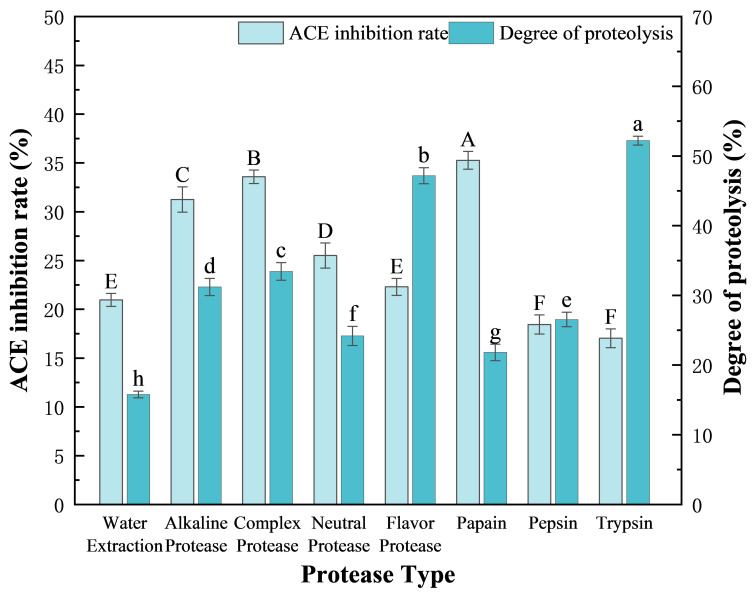
Effects of the seven proteases on the DH and ACE inhibition rates of the obtained peptides. The concentration of crude peptide solution was 2 g/100 g. Duncan’s test was used. When *p* < 0.05, different capital letters (A–F) represent significant differences in ACE inhibition rate, different lowercase letters (a–h) represent significant differences in the degree of hydrolysis.

Owing to the specificity of their recognition sites, different proteases yielded diverse active peptides during the degradation of *F. velutipes* protein, which directly affected the ACE inhibitory activity of the peptides. To identify the optimal protease for the enzymatic hydrolysis of *F. velutipes* protein, a total of seven proteases were screened, and the DH and ACE inhibition rate of the resulting peptides were determined (Figure 2). The results showed that all the seven proteases (4000 U/g) could hydrolyze the *F. velutipes* protein at varying degrees, and that the resulting peptides (2 g/100 g) exhibited ACE inhibitory effects. When compared with peptides obtained using the water extraction method without protease, those extracted using protease presented a significantly improved ACE inhibition rate. Trypsin has high catalytic efficiency and can degrade the protein into a large number of peptides. Flavor protease is mainly composed of endoprotease and exoprotease, and the synergistic effect significantly improves the degree of hydrolysis. However, excessive hydrolysis will destroy the structure of functional peptides, so the ACE inhibition rate of peptides hydrolyzed by trypsin and flavor protease is low. Peptides derived using papain and complex protease showed moderate DH value rates (21.82 ± 1.18% and 33.44 ± 1.25%, respectively), but high ACE inhibition (35.28 ± 0.91% and 33.59 ± 0.69%, respectively), when compared with those obtained using flavor protease and trypsin (DH values: 47.18 ± 1.16% and 52.21 ± 0.64%, respectively; *p* < 0.05). Therefore, papain:complex protease (the amount of enzyme added is 4000 U/g) at a ratio of 1:2 (determined from previous experiments) was selected as the optimal enzyme preparations mixture for the production of ACEI peptides from *F. velutipes* protein by double-enzymatic hydrolysis.

### 3.2. Optimization of Process Parameters for the Production of High-Activity ACE Inhibitory Peptides by Double-Enzymatic Hydrolysis Method

Based on previous results, the three factors of the enzymolysis time (*A*), enzymolysis temperature (*B*), and enzymolysis pH (*C*) were used as independent variables, and the ACE inhibition rate was employed as the response value to determine the optimal test conditions (Table 3 and Table 4).

Design Expert 8.0.6 software was utilized to assess the response surface test results and quadratic multiple regression fit (Table 3). The quadratic regression equation was obtained as follows: Y = 66.44 + 0.72 × *A* − 1.58 × *B* − 1.37 × *C* + 1.89 × *A* × *B* + 1.72 × *A* × *C* − 0.32 × *B* × *C* − 8.74 × *A*^2^ − 11.38 × *B*^2^ − 10.29 × *C*^2^, where Y is the ACE inhibition rate (%), *A* is the enzymolysis time, *B* is the enzymolysis temperature, and *C* is the enzymolysis pH.

As can be seen from Table 4, the model is extremely significant (*p* < 0.001), the coefficient of determination R^2^ of the model is 0.9973, and the lack of fit is not significant (0.7224 > 0.05). Therefore, the model can be used to analyze the test results. The enzymolysis time, enzymolysis temperature, and enzymolysis pH were all significant, and the order of significance was enzymolysis temperature (*B*) > enzymolysis pH (*C*) > enzymolysis time (*A*). Therefore, the enzymolysis temperature had the greatest effect on the ACE inhibition rate of *F. velutipes*, followed by the enzymolysis pH, and the enzymolysis time had the least effect.

**Figure 3:**
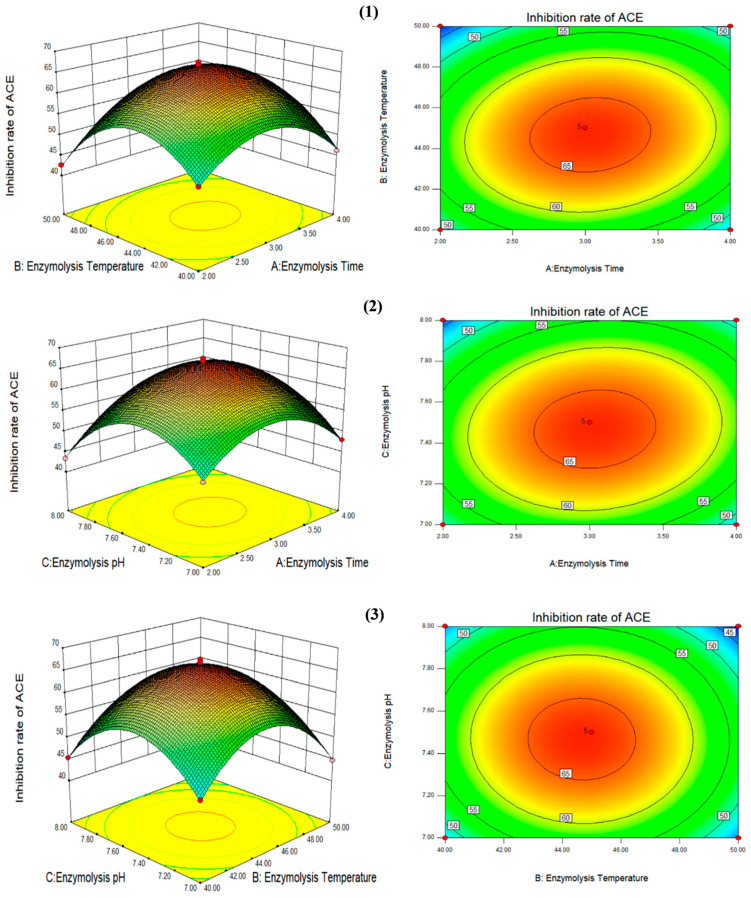
Curved surface diagram of the effect of the three factors on the ACE inhibition rates. (**1**) The interaction between enzymolysis time (*A*) and enzymolysis temperature (*B*); (**2**) the interaction between enzymolysis pH (*C*) and enzymolysis time (*A*); (**3**) the interaction between enzymolysis pH (*C*) and enzymolysis temperature (*B*) was expressed. Picture on the left: the red points represent that design points above predicted value; the pink points represent that design points below predicted value. Picture on the right: the closer to the central area (orange red), the higher the inhibition rate of ACE ; the closer to the edge area (blue), the lower the inhibition rate of ACE.

As shown in Table 4 and Figure 3, the change curves of the enzymolysis time and enzymolysis temperature were steeper than those of the enzymolysis time and enzymolysis pH, indicating that the effects of the enzymolysis time and enzymolysis temperature on the ACE inhibition rate of *F. velutipes* polypeptide were more significant than those of the enzymolysis time and enzymolysis pH. In contrast, the interaction between the enzymolysis temperature and enzymolysis pH had no significant effect on the ACE inhibition rate of the peptides. The theoretical optimum enzymolysis conditions predicted by response surface analysis software were as follows: enzymolysis time, 3.22 h; enzymolysis temperature, 42.88 °C; enzymolysis pH, 7.27; and ACE inhibition rate, 62.82%. However, considering the practicality of the experiment, the enzymatic hydrolysis conditions were adjusted as follows: enzymolysis time, 3.2 h; enzymolysis temperature, 43 °C; and enzymolysis pH, 7.3. The ACE inhibition rate of the peptides derived from *F. velutipes* protein under the adjusted enzymatic hydrolysis conditions was 63.57%, indicating that this model can better reflect the conditions of protein enzymatic hydrolysis.

### 3.3. Separation and Purification

#### 3.3.1. Screening of Macroporous Resin

**Figure 4:**
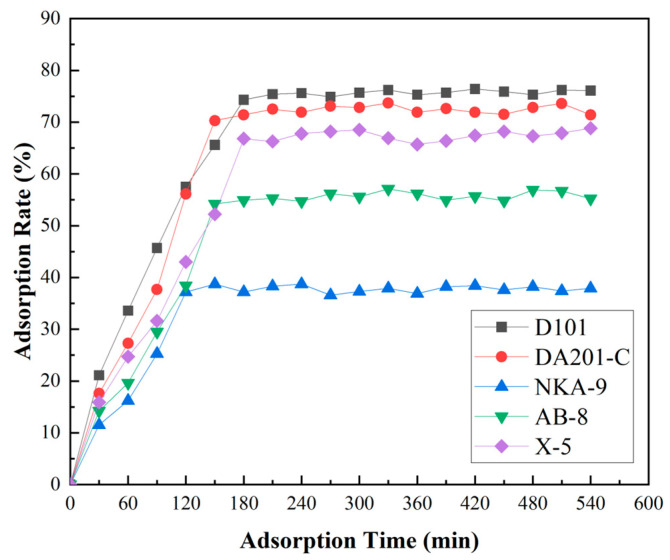
Static adsorption curves of the five macroporous resins. D101, DA201-C, X-5, AB-8, and NKA-9 are different types of macroporous resins, respectively. Samples were taken every 30 min, and samples were collected at 18 time points (30–540 min) within 9 h.

The adsorption rate of five resins on ACE inhibitory peptides (30 mg/mL) of *F. velutipes* is shown in Figure 4. The adsorption effect of resins from strong to weak is D101, DA201-C, X-5, AB-8, and NKA-9. Among them, the adsorption effect of D101 was better than that of other types of resins, and it began to reach equilibrium at 180 min, and the adsorption rate was 74.3%. It shows that D101 had strong adsorption for *F. velutipes* ACE inhibitory peptides. Therefore, D101 macroporous resin was selected as the most suitable resin for the separation and purification of ACEI peptides from *F. velutipes*.

#### 3.3.2. Purification of ACE Inhibitory Peptides by Gel Chromatography

**Figure 5:**
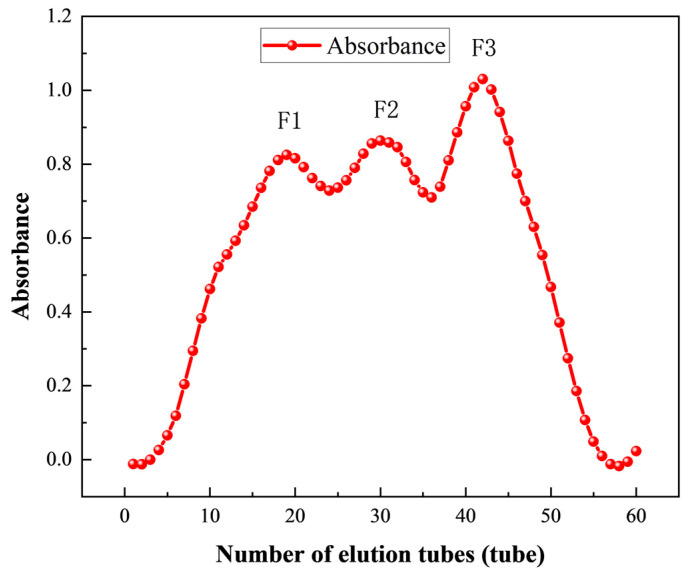
Absorbance of Sephadex G-100 fractions.

Sephadex G-100 was used to further purify the peptide solution (50 mg/mL) obtained after separation by macroporous resin. As shown in Figure 5, the eluent of the first 60 tubes was collected, and the ACE inhibitory peptides were separated by Sephadex G-100 to obtain three different peaks: F1 (tube 19), F2 (tube 31), and F3 (tube 42). The ACE inhibition rates of F1, F2, and F3 were 57.19%, 64.24%, and 79.51%, respectively, with F3 presenting a significantly better ACE inhibition rate than F1 and F2. Therefore, the eluent of F3 (tube 42) was freeze-dried and stored for subsequent RP-HPLC separation.

#### 3.3.3. Molecular Weight Distribution of the ACE Inhibitory Peptides Before and After Purification

**Figure 6:**
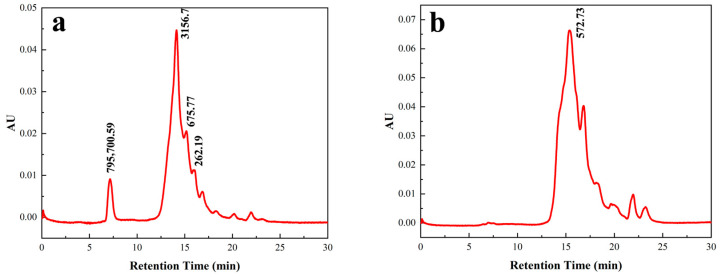
Molecular weight chromatograms of peptides (**a**) before and (**b**) after purification.

The peptides with high ACE inhibitory activity were mainly those with lower molecular weights [34,35]. As shown in Figure 6, the molecular weight range of the crude *F. velutipes* peptide solution before purification was 145.06–795,700.59 Da, with peptides of molecular weights < 675.77 Da accounting for 26.26% of the sample. In contrast, the molecular weight range of the purified peptide was 142.49–2000.148 Da, with peptides of molecular weights < 572.73 Da constituting 68.83% of the sample. These results indicated that macroporous resin D101 and Sephadex G-100 gel chromatography demonstrated superior separation and purification effects on the peptide, yielding a higher number of low-molecular-weight peptides, predominantly comprising short peptides with potential biological activity.

#### 3.3.4. Purification by RP-HPLC

**Figure 7:**
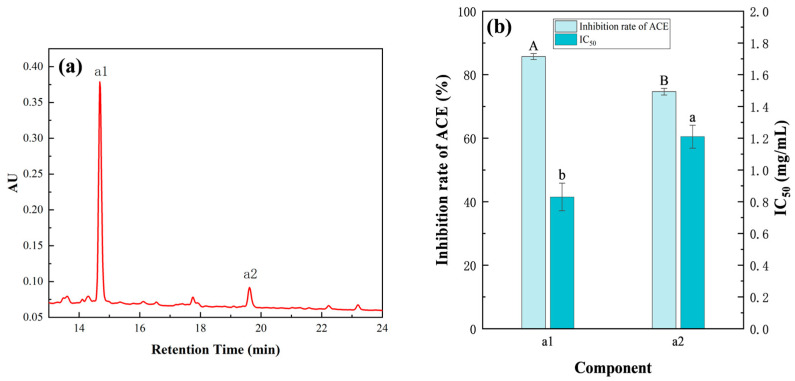
Active components and activity analysis of peptides purified by RP-HPLC. (**a**) RP-HPLC chromatogram of peptide. (**b**) ACE inhibition rates and IC_50_ values of a1 and a2. Duncan’s test was used. When *p* < 0.05, different capital letters (A,B) represent significant differences in ACE inhibition rate, different lowercase letters (a,b) represent significant differences in IC_50_. The concentrations of a1 and a2 were 2 mg/mL.

The F3 fraction (2 mg/mL) was separated by RP-HPLC, which yielded two subcomponents, namely, a1 and a2 (Figure 7a). The ACE inhibition rates of the a1 and a2 components were 85.73 ± 0.95% and 74.67 ± 1.05%, with corresponding IC_50_ values of 0.83 ± 0.09 and 1.21 ± 0.07 mg/mL, respectively, indicating that RP-HPLC had a better separation effect on components (Figure 7b). As the IC_50_ value of a1 was lower than that of a2, a1 was selected for subsequent peptide sequence identification.

#### 3.3.5. Comparison of ACE Inhibitory Activities of the Peptides Before and After Isolation and Purification

**Figure 8:**
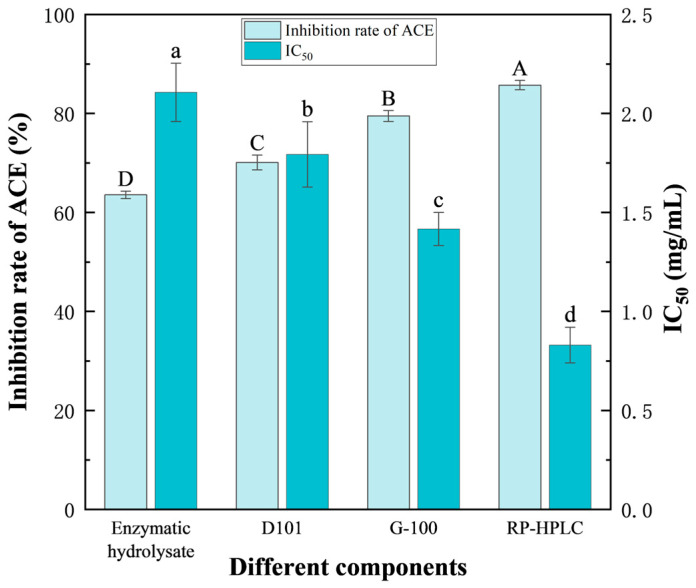
Changes in the ACE inhibitory activity and IC_50_ value of the peptides before and after separation and purification. Enzymatic hydrolysate indicates the product of enzymatic hydrolysis, D101 represents the component with the highest activity after macroporous resin separation and purification, G-100 denotes the component with the highest activity after gel chromatography separation and purification, and RP-HPLC refers to the component with the highest activity after RP-HPLC separation and purification. Duncan’s test was used. When *p* < 0.05, different capital letters (A–D) represent significant differences in the ACE inhibition rate, different lowercase letters (a–d) represent significant differences in IC_50_. The concentrations of enzymatic hydrolysate were 2 g/100 g; the concentrations of components separated from D101 were 30 mg/mL; the concentrations of components separated from G-100 were 50 mg/mL; the concentrations of components separated from RP-HPLC were 2 mg/mL.

As shown in Figure 8, with gradual separation and purification, the ACE inhibitory activity of the prepared peptides increased, while the corresponding IC_50_ value decreased. The ACE inhibition rate of the *F. velutipes* protein hydrolysate was 63.57 ± 0.76% (IC_50_ = 2.11 ± 0.15 mg/mL). However, following D101 macroporous resin separation, Sephadex G-100 gel chromatography, and RP-HPLC separation and purification, the ACE inhibition rates of the peptides increased to 70.10 ± 1.49% (IC_50_ = 1.79 ± 0.17 mg/mL), 79.51 ± 1.11% (IC_50_ = 1.42 ± 0.08 mg/mL), and 85.73 ± 0.95% (IC_50_ = 0.83 ± 0.09 mg/mL), respectively, exhibiting a 22.91% increase, when compared with that of the initial enzymatic hydrolysate. These findings suggested that the optimized conditions were feasible for the production of ACE inhibitory peptides from *F. velutipes*.

### 3.4. Screening and Identification of ACE Inhibitory Peptides Derived from F. velutipes

**Figure 9:**
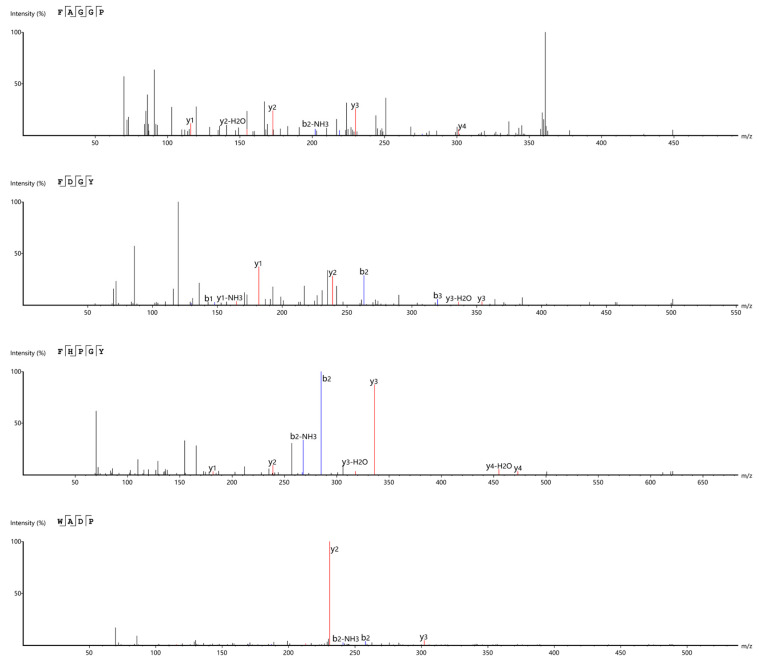
Secondary mass spectra of the four peptides derived from *F. velutipes* protein.

LC-MS/MS was used to sequence the a1 component (after RP-HPLC purification). A total of 390 peptides with a de novo score of 95 were selected; the activity was predicted by the Peptide Ranker. Subsequently, 20 peptides that met the specified criteria were chosen following a screening process against relevant websites (see Section 2.10) and subjected to molecular docking. Finally, considering the physical and chemical properties such as the binding energy, hydrogen bond, and hydrophobic force, four novel peptides, namely, FAGGP, FDGY, FHPGY, and WADP, with potential ACE inhibitory activity were screened out. It must be noted that the active sites of ACE cannot usually accommodate large-molecular-weight peptides; consequently, most of the peptide sequences with antihypertensive activity are relatively short, typically containing 2–12 amino acid residues. Thus, the sequence length and structural characteristics of the four peptides FAGGP, FDGY, FHPGY, and WADP suggest their potential ACE inhibitory activity. The secondary mass spectra of these four peptides are shown in (Figure 9). The four peptides screened by the Peptide Ranker in this study contain His, Phe, Tyr, Trp, and Arg, which are the general characteristics of ACE inhibitory peptides [36].

### 3.5. Molecular Docking Analysis

**Figure 10:**
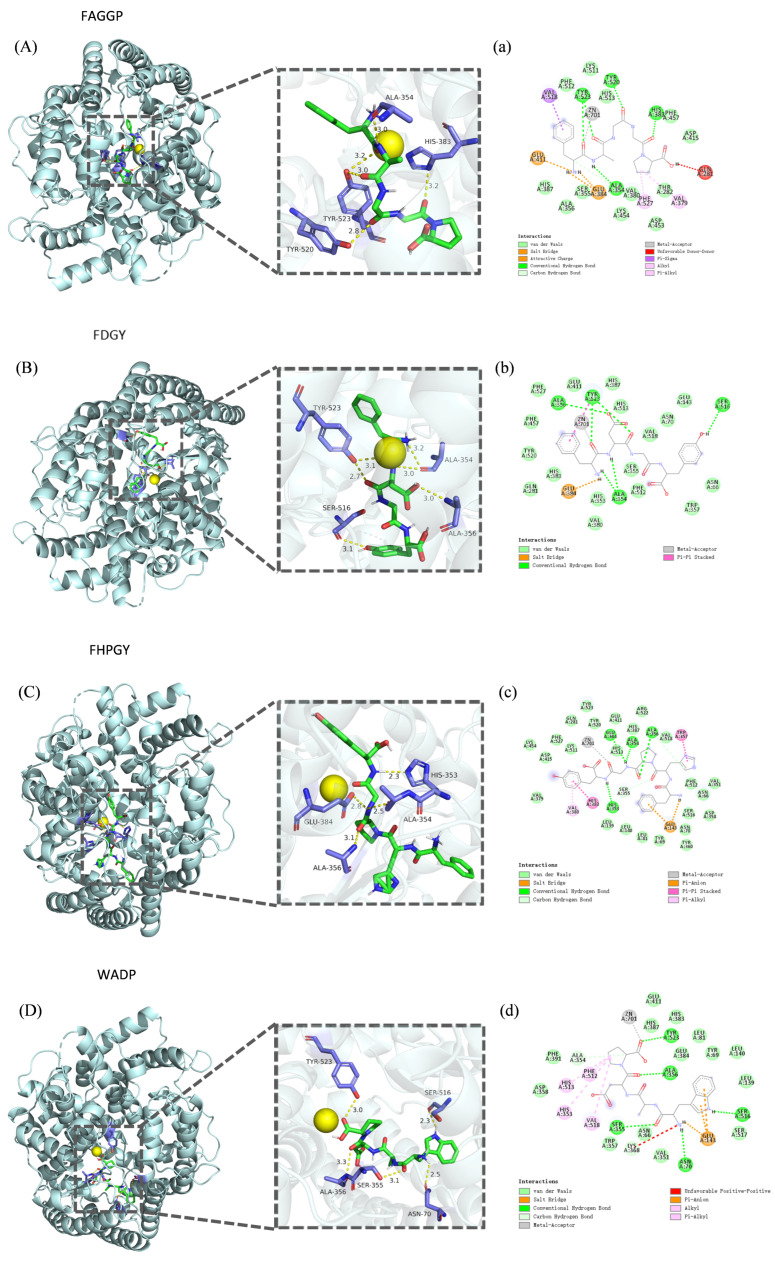
Molecular docking of ACE receptor with four active peptides derived from *F. velutipes* protein. (**A**–**D**) The 3D maps of the four peptides binding to the ACE receptor. (**a**–**d**) The 2D maps of the four peptides binding to the key sites of the ACE receptor. (**A**) and (**a**): Binding of peptide FAGGP to the ACE receptor. (**B**) and (**b**): Binding of peptide FDGY to the ACE receptor. (**C**) and (**c**): Binding of peptide FHPGY to the ACE receptor. (**D**) and (**d**): Binding of peptide WADP to the ACE receptor.

The binding mode between ligand and receptor can be predicted by molecular docking, and many studies have employed molecular docking methods to analyze the interaction between ACE and various peptides [37,38]. In the present study, the binding energies of the four peptides FAGGP, FDGY, FHPGY, and WADP were found to be −9.5, −9.4, −10.3, and −9.5 kcal/mol, respectively, indicating that these peptides had good affinity with the ACE active sites, consistent with the results reported in previous works [39,40,41]. As shown in Figure 10 and Table 5, peptides FAGGP and FDGY formed three and four hydrogen bonds with the residues Ala354 and Tyr523 in the S1 pocket of the ACE active site, respectively; peptide FHPGY formed two hydrogen bonds with the residues Glu384 and Ala354 in the S1 pocket of the ACE active site; and peptide WADP formed a hydrogen bond with the residue Tyr523 in the S1 pocket of the ACE active site. Furthermore, peptide FAGGP formed a hydrogen bond with the residue Tyr520 in the S2 pocket of the ACE active site (His513, His353, Tyr520, Lys511, and Gln281) and a hydrogen bond with residue His383 in the HEXXH zinc-coordinating motif (His387, His383, Glu411). In addition, peptide FHPGY formed a hydrogen bond with the residue His353 in the S2 pocket of the ACE active site, while peptides FAGGP, FHPGY, and WADP exhibited a metal–acceptor interaction with Zn701, respectively.

It must be noted that S1, S2, and S′ (Glu162) are the three active pockets of the ACE active site. In addition, ACE also contains an HEXXH (His-Glu-X-X-His) zinc-coordinating motif, and can interact with the zinc-binding domain [37,42]. The metal–acceptor interaction has been reported to be a key factor in ACE inhibitory activity, and the formation of a hydrogen bond is considered to be one of the most important non-covalent interactions in the binding process of potential ACE inhibitory peptides to ACE [43,44].

### 3.6. Validation of ACE Inhibitory Activity of the Selected Peptides

The ACE inhibitory activity of the four synthesized peptides, FAGGP, FDGY, FHPGY, and WADP, was evaluated by measuring their ACE inhibition rates with captopril as the positive control (Table 6). Peptide FHPGY exhibited the highest ACE inhibition rate (91.41 ± 1.01%), followed by peptides FAGGP, WADP, and FDGY (85.92 ± 1.81%, 83.04 ± 0.68%, and 80.48 ± 0.84%, respectively). The inhibitory effects reached 92.60%, 87.04%, 84.13%, and 81.53% of the positive control, respectively. Furthermore, the IC_50_ values for the peptides FHPGY, FAGGP, WADP, and FDGY were 14.79 ± 0.34, 29.17 ± 1.37, 41.27 ± 0.70, and 91.55 ± 1.09 µM, respectively, indicating the significant ACE inhibitory ability of these peptides, which are lower or comparable to those reported in the literature [45,46,47]. Despite their higher IC_50_ values, when compared with that for captopril (0.01 µM), the peptides screened in this study were derived from the fruiting body of natural edible fungi, with advantages including the absence of toxic side effects and enhanced safety. It has the value of developing functional food for lowering blood pressure.

## 4. Discussion

Bioactive peptides have attracted extensive attention in the scientific community because of their small molecular weight, high safety, and easy absorption and digestion by the human body [48]. Moreover, in terms of the development of hypertension control drugs, the substitution of food-derived ACE inhibitory peptides for chemical synthesis inhibitors has become a development trend in this field [49,50].

In recent years, with the popularization of the industrialized production technology of edible fungi, there have been signs of overcapacity in the *F. velutipes* industry. *F. velutipes* is an ideal raw material for the production of antihypertensive peptides because of its low price and much higher protein content than most plants [29]. Compared with chemical synthetic drugs, *F. velutipes* ACE inhibitory peptides have the advantages of high safety and non-toxic side effects. ACE inhibitory peptides from *F. velutipes* are almost non-allergenic with other natural sources of ACE inhibitory peptides such as soybeans, squid, etc. [20,49,51]. The results of this study showed that the polypeptide activity obtained by double-enzyme digestion was better than that of single-enzyme digestion, which was consistent with other reported results. However, it should be noted that the reaction conditions of proteases should be controlled. In this study, trypsin and flavor proteases had a higher degree of hydrolysis of ACE inhibitory peptides and a lower ACE inhibition rate, which may be due to excessive hydrolysis to destroy the structure of bioactive peptides.

The binding mode between ligand and receptor can be predicted by molecular docking [37]. The active sites of ACE are usually unable to accommodate large-molecular-weight peptides, and most of the peptide sequences with antihypertensive activity are relatively short [36]. Studies have shown that potential ACE inhibitory peptides are usually composed of 4 to 6 amino acids, and peptides containing Tyr, His, Phe, Arg, and Trp are the general characteristics of ACEI peptides [36,52]. The four new peptides (FAGGP, FDGY, FHPGY, WADP) screened in this study were consistent with the above characteristics. The sequence length and structural characteristics of these four peptides make them potential ACE inhibitors.

## 5. Conclusions

In this study, the optimal conditions for the preparation of ACE inhibitory peptides from *F. velutipes* protein by double-enzymatic hydrolysis were obtained as follows: enzymatic hydrolysis time 3.2 h, enzymatic hydrolysis temperature 43 °C, and enzymatic hydrolysis pH 7.3. Under this condition, the ACE inhibition rate was 62.82%. After screening, four new peptides (FAGGP, FDGY, FHPGY, WADP) were found. The molecular docking results showed that the four peptides formed hydrogen bonds with the S1 pocket, S2 pocket, and HEXXH zinc-binding motif of ACE, indicating that these four peptides had good affinity with the active site of ACE. The activities of the four synthesized peptides reached 92.60%, 87.04%, 84.13%, and 81.53% of the positive control captopril, respectively, suggesting that these peptides have the potential to develop new antihypertensive products. Our study confirmed that *F. velutipes* can be used as a good source of food-borne ACE inhibitory peptides, which not only provides a new way for the high-value-added utilization of *F. velutipes*, but also provides a scientific basis for the development of new antihypertensive drugs or functional foods.

## Figures and Tables

**Table 2:** Coding values of the response surface test factor levels.

Level	Factor		
	*A* Enzymolysis Time /h	*B* Enzymolysis Temperature/°C	*C* Enzymolysis pH
−1	2	40	7
0	3	45	7.5
1	4	50	8

**Table 3:** Response surface test results.

Serial Number	*A* Enzymolysis Time /h	*B* Enzymolysis Temperature /°C	*C* Enzymolysis pH	Inhibition Rate of ACE /%
1	−1	0	−1	49.38
2	−1	1	0	42.61
3	0	1	−1	44.78
4	0	0	0	66.61
5	0	1	1	41.26
6	0	0	0	65.39
7	1	0	1	48.87
8	0	−1	−1	47.63
9	−1	0	1	43.36
10	0	0	0	65.73
11	0	0	0	66.92
12	1	0	−1	48.02
13	−1	−1	0	49.22
14	0	−1	1	45.39
15	1	−1	0	46.25
16	1	1	0	47.19
17	0	0	0	67.53

**Table 4:** Analysis of variance.

Source	Sum of Squares	Degree of Freedom	Mean Square	F Value	*p* Value	Significance
Model	1530.17	9	170.02	288.98	<0.0001	**
*A* (enzymolysis time)	4.15	1	4.15	7.05	0.0327	*
*B* (enzymolysis temperature)	20.00	1	20.00	34.00	0.0006	**
*C* (enzymolysis pH)	14.93	1	14.93	25.38	0.0015	*
*AB*	14.25	1	14.25	24.22	0.0017	*
*AC*	11.80	1	11.80	20.06	0.0029	*
*BC*	0.41	1	0.41	0.70	0.4316	
*A* ^2^	321.48	1	321.48	546.42	<0.0001	**
*B* ^2^	545.33	1	545.33	926.89	<0.0001	**
*C* ^2^	445.87	1	445.87	757.84	<0.0001	**
Residual error	4.12	7	0.59			
Lack of fit	1.06	3	0.35	0.46	0.7224	
Pure error	3.05	4	0.76			
Cor total	1534.29	16				

Note: Using ‘**’ to represent the extremely significant level (*p* < 0.001); the significance level was expressed as ‘*’ (*p* < 0.05).

**Table 5:** Molecular docking of the synthesized peptides with ACE.

Peptide Sequence	Binding Energy (kcal/mol)	Conventional Hydrogen Bond	Van der Waals Interaction	Carbon Hydrogen Bond	Other Interactions
FAGGP	−9.5	Tyr523, Tyr520, Ala354, His383	His387, Ser355, Lys454, Thr282, Val380, Asp453, Phe512, His513, Lys511, Phe457, Asp415, Ala356		Zn^2+^, Val518, Glu411, Glu384, Gln281, Phe527, Val379
FDGY	−9.4	Ala354, Ser516, Ala356, Tyr523	Val380, His353, His383, Gln281, Tyr520, Phe457, Phe527, Glu411, His387, Ser355, Phe512, His513, Trp357, Asn66, Val518, Glu143, Asn70		Zn^2+^, Glu384
FHPGY	−10.3	Glu384, Ala354, His353, Ala356	Lys454, Asp415, Val379, Phe527, Lys511, Gln281, Tyr523, Tyr520, His513, Leu139, Glu411, Leu140, His387, Arg522, Leu81, Tyr69, Val518, Tyr360, Asn70, Phe512, Val351, Ser516, Asp358, Asn66	Ser355	Zn^2+^, His383, Glu143, Trp357, Val380
WADP	−9.5	Ser355, Ala356, Tyr523, Asn70, Ser516	Glu411, His387, His383, Leu81, Tyr69, Leu140, Leu139, Ser517, Val351, Asn66, Phe391, Asp358, Glu384, Trp357	Ala354, Lys368	Zn^2+^, Glu143, Phe512, His513, His353, Val518

**Table 6:** ACE inhibition rates and IC_50_ values for the synthesized peptides.

Peptide Sequence	Mw (Da)	Inhibition Rate of ACE (%)	IC_50_ (µM)
FAGGP	433.46	85.92 ± 1.81 ^c^	29.17 ± 1.37 ^c^
FDGY	447.48	80.48 ± 0.84 ^e^	91.55 ± 1.09 ^a^
FHPGY	487.51	91.41 ± 1.01 ^b^	14.79 ± 0.34 ^d^
WADP	619.68	83.04 ± 0.68 ^d^	41.27 ± 0.70 ^b^
Captopril		98.64 ± 0.73 ^a^	0.01

Duncan’s test was used. When *p* < 0.05, different lowercase letters (a–e) represent significant differences.

## Data Availability

The original contributions presented in this study are included in the article. Further inquiries can be directed to the corresponding author.

## Abbreviations

The following abbreviations are used in this manuscript:

ACE	Angiotensin I-converting enzyme
ACEI	Angiotensin I-converting enzyme inhibitor
DH	The degree of hydrolysis
DTT	Dithiothreitol
FAPGG	N-[3-(2-furylacryloyl)]-L-phenylalanyl-glycyl-glycine
IAM	Iodoacetamide
LC-MS/MS	Liquid chromatography–tandem mass spectrometry
HEXXH	His-Glu-X-X-His
HPLC	High-performance liquid chromatography
RP-HPLC	Reversed-phase high-performance liquid chromatography

## References

[1] Sun X., Acquah C., Aluko R., Udenigwe C. (2020). Considering food matrix and gastrointestinal effects in enhancing bioactive peptide absorption and bio-availability. J. Funct. Foods.

[2] Jahandideh F., Liu P., Wu J. (2018). Purification and identifi-cation of adipogenic-differentiating peptides from egg white hydrolysate. Food Chem..

[3] Elisha C., Bhagwat P., Pillai S. (2024). Emerging production techniques and potential health promoting properties of plant and animal protein-derived bioactive peptides. Crit. Rev. Food Sci..

[4] Benson L.N., Guo Y., Katherine D., Christoph M., Liu Y., Mu S. (2023). The link between immunity and hypertension in the kidney and heart. Front. Cardiovasc. Med..

[5] Duan X., Dong Y., Zhang M., Li Z., Bu G., Chen F. (2023). Identification and molecular interactions of novel ACE inhibitory peptides from rapeseed protein. Food Chem..

[6] Fadimu G.J., Gan C.Y., Olalere O.A., Farahnaky A., Gill H., Truong T. (2023). Novel antihypertensive peptides from lupin protein hydrolysate: An in-silico identification and molecular docking studies. Food Chem..

[7] Ma K., Wang Y., Wang M., Wang Z., Wang X., Ju X., He R. (2021). Antihypertensive activity of the ACE–renin inhibitory peptide derived from Moringa oleifera protein. Food Funct..

[8] Zhang A., Yang Y., Huo X., Long P., Zheng Y., Guo X., Liu J., Zhang Y. (2025). Multifunctional ACE-inhibitory peptides with antioxidant and ferrous-chelating capacities from ginkgo kernel glutelin-2 hydrolysates: Identification, virtual screening, inhibition mechanism, and gastrointestinal stability studies. LWT-Food Sci. Technol..

[9] Du T., Xu Y., Xu X., Xiong S., Zhang L., Dong B., Huang J., Huang T., Xiao M., Xiong T. (2023). ACE inhibitory peptides from enzymatic hydrolysate of fermented black sesame seed: Random forest-based optimization, screening, and molecular docking analysis. Food Chem..

[10] Cutrell S., Alhomoud I.S., Mehta A., Talasaz A.H., Van Tassell B., Dixon D.L. (2023). ACE-inhibitors in hypertension: A historical perspective and current insights. Curr. Hypertens. Rep..

[11] Memarpoor-Yazdi M., Asoodeh A., Chamani J. (2012). Structure and ace-inhibitory activity of peptides derived from hen egg White Lysozyme. Int. J. Pept. Res. Ther..

[12] Du T., Huang J., Xu X., Xiong S., Zhang L., Xu Y., Zhao X., Huang T., Xiao M., Xiong T. (2024). Effects of fermentation with Lactiplantibacillus plantarum NCU116 on the antihypertensive activity and protein structure of black sesame seed. Int. J. Biol. Macromol..

[13] Das M., Gangopadhyay A., Saha A., Dhar P. (2024). Novel ACE inhibitory peptides from enzymatic hydrolysate of Channa punctata protein: In vitro and In silico assay of structure-activity relationship. Food Biosci..

[14] Yu D., Qing H., Jing K., Yu Q., Chang F., Wang B. (2023). Angiotensin-I-Converting Enzyme (ACE)-Inhibitory Peptides from the Collagens of Monkfish (*Lophius litulon*) Swim Bladders: Isolation, Characterization, Molecular Docking Analysis and Activity Evaluation. Mar. Drugs.

[15] Du T., Yang J., Qin Y., Huang X., Li J., Xiong S., Xu X., Zhang L., Zhao M., Li H. (2025). Transport and action of sesame protein-derived ACE inhibitory peptides ITAPHW and IRPNGL. Food Chem..

[16] Zheng Y., Wang X., Zhuang Y., Li Y., Tian H., Shi P., Li G. (2019). Isolation of Novel ACE-Inhibitory and Antioxidant Peptides from Quinoa Bran Albumin Assisted with an In Silico Approach: Characterization, In Vivo Antihypertension, and Molecular Docking. Molecules.

[17] Sahingil D., Gokce Y., Celikbicak O., Hayaloglu A.A. (2022). ACE-inhibitory activities of peptide fractions (<3 kDa) and identification of peptide sequence by MALDI-ToF-MS in model cheeses incorporating different Lactobacillus species. J. Food Compos. Anal..

[18] Chen B., Wang X., Zhang J., Wang L. (2024). Peptidomics-based study of antihypertensive activity: Discovery of novel ACE inhibiting peptides from peanut yogurt. Food Funct..

[19] Chan P., Matanjun P., Budiman C., Shapawi R., Lee J.S. (2025). Valorisation of Hybrid Grouper Bone Gelatine: Identification and In Silico Analysis of Novel ACE-Inhibitory and Antioxidant Peptides Obtained Via Ultrafiltration. Waste Biomass Valorization.

[20] Li M., Liang Q., Zhang Y., Jiang X., Gu Y., Song X., Wang X., Shi W. (2025). Screening of Potential Angiotensin-Converting Enzyme-Inhibitory Peptides in Squid (*Todarodes pacificus*) Skin Hydrolysates: Preliminary Study of Its Mechanism of Inhibition. Mar. Drugs.

[21] Juhui C., Hwan K.S., Jin H.K., Taek J.H., Mooha L., Cheorun J. (2019). Isolation and identification of angiotensin I-converting enzyme inhibitory peptides derived from thermolysin-injected beef *M. longissimus*. Asian-Australas. J. Anim. Sci..

[22] Han X., Jia Y.Q., Liu C.Y., Wang H.Y., Zhu Z.Y. (2023). Structure identification and inhibitor y mechanism evaluation of three novel angiotensin converting enzyme (ACE) inhibitory peptides from small-aroma chicken. Process Biochem..

[23] Wang R., Yun J., Wang S., Bi Y., Zhao F. (2022). Optimisation and Characterisation of Novel Angiotensin-Converting Enzyme Inhibitory Peptides Prepared by Double Enzymatic Hydrolysis from *Agaricus bisporus* Scraps. Foods.

[24] Paisansak S., Sangtanoo P., Srimongkol P., Saisavoey T., Reamtong O., Choowongkomon K., Karnchanatat A. (2020). Angiotensin-I converting enzyme inhibitory peptide derived from the shiitake mushroom (*Lentinula edodes*). J. Food Sci. Technol..

[25] Shi J., Yang Z., Xu M., Zhao G., Gao Y., Zheng H., Feng J. (2025). Structure characterization and mechanism of angiotensin I-converting enzyme (ACE) inhibitory peptides modified by plastein reaction derived from tiger nut (*Cyperus esculentus*). Front. Sustain. Food Syst..

[26] Kimatu M.B., Zhao L., Biao Y., Ma G., Yang W., Pei F., Hu Q. (2017). Antioxidant potential of edible mushroom (*Agaricus bisporus*) protein hydrolysates and their ultrafiltration fractions. Food Chem..

[27] Ramos I.R., Delgado-Andrade C. (2017). The beneficial role of edible mushrooms in human health. Curr. Opin. Food Sci..

[28] Tang C., Hoo P.C.X., Tan L.T.H., Pusparajah P., Khan T.M., Lee L.H., Goh B.H., Chan K.G. (2016). Golden needle mushroom: A culinary medicine with evidenced-based biological activities and health promoting properties. Front. Pharmacol..

[29] Wang Y., Zhang H. (2021). Advances in the extraction, purification, structural-property relationships and bioactive molecular mechanism of *Flammulina velutipes* polysaccharides: A review. Int. J. Biol. Macromol..

[30] Attaran Dowom S., Rezaeian S., Pourianfar H.R. (2019). [M41] Agronomic and environmental factors affecting cultivation of the winter mushroom or Enokitake: Achievements and prospects. Appl. Microbiol. Biotechnol..

[31] Cui C., Hu Q., Ren J., Zhao H., You L., Zhao M. (2013). Effect of the structural features of hydrochloric acid-deamidated wheat gluten on its susceptibility to enzymatic hydrolysis. J Agric. Food Chem..

[32] Wang F., Zhou B. (2020). Investigation of angiotensin-I-converting enzyme (ACE) inhibitory tri-peptides: A combination of 3D-QSAR and molecular docking simulations. RSC Adv..

[33] Mooney C., Haslam N.J., Pollastri G., Shields D.C. (2012). Towards the improved discovery and design of functional peptides: Common features of diverse classes permit generalized prediction of bioactivity. PLoS ONE.

[34] Hernández-Ledesma B., Contreras M.D.M., Recio I. (2010). Antihypertensive peptides: Production, bioavailability and incorporation into foods. Adv. Colloid Interface Sci..

[35] Alemán A., Gómez-Guillén M.C., Montero P. (2013). Identification of ace-inhibitory peptides from squid skin collagen after in vitro gastrointestinal digestion. Food Res. Int..

[36] Ma M., Feng Y., Miao Y., Shen Q., Tang S., Dong J., Zhang J., Zhang L. (2023). Revealing the Sequence Characteristics and Molecular Mechanisms of ACE Inhibitory Peptides by Comprehensive Characterization of 160,000 Tetrapeptides. Foods.

[37] Chen J., Yu X., Chen Q., Wu Q., He Q. (2022). Screening and mechanisms of novel angiotensin-I-converting enzyme inhibitory peptides from rabbit meat proteins: A combined in silico and in vitro study. Food Chem..

[38] Xu Z., Wu C., Sun-Waterhouse D.X., Zhao T., Waterhouse G.I.N., Zhao M., Su G. (2021). Identification of post-digestion angiotensin-I converting enzyme (ACE) inhibitory peptides from soybean protein Isolate: Their production conditions and in silico molecular docking with ACE. Food Chem..

[39] Chen L., Cheng F., Chen H., Shu G. (2024). Preparation and identification of novel angiotensin-I-converting enzyme inhibitory peptides from Moringa oleifera leaf. LWT-Food Sci. Technol..

[40] Lin Z., Lai J., He P., Pan L., Zhang Y., Zhang M., Wu H. (2023). Screening, ACE-inhibitory mechanism and structure-activity relationship of a novel ACE-inhibitory peptide from *Lepidium meyenii* (Maca) protein hydrolysate. Food Biosci..

[41] Chanajon P., Hamzeh A., Tian F., Roytrakul S., Oluwagunwa A.O., Kadam D., Aluko E.R., Aueviriyavit S., Wongwanakul R., Yongsawatdigul J. (2024). Hypotensive effect of potent angiotensin-I-converting enzyme inhibitory peptides from corn gluten meal hydrolysate: Gastrointestinal digestion and transepithelial transportation modifications. Food Chem..

[42] Pan D., Cao J., Guo H., Zhao B. (2012). Studies on purification and the molecular mechanism of a novel ACE inhibitory peptide from whey protein hydrolysate. Food Chem..

[43] René T., Christensen M.H. (2006). A new technique for high-accuracy molecular docking. J. Med. Chem..

[44] Glowacki E.D., Irimia-Vladu M., Bauer S., Sariciftci N.S. (2013). Hydrogen-bonds in molecular solids from biological systems to organic electronics. J. Mater. Chem. B..

[45] Xiang H., Huang H., Shao Y., Hao S., Li L., Wei Y., Chen S., Zhao Y. (2024). Angiotensin-I-converting enzyme inhibitory peptides from eel (*Anguilla japonica*) bone collagen: Preparation, identification, molecular docking, and protective function on HUVECs. Front. Nutr..

[46] Zou L., Zhou Y., Yu X., Chen C., Xiao G. (2023). Angiotensin I-Converting Enzyme Inhibitory Activity of Two Peptides Derived from In Vitro Digestion Products of Pork Sausage with Partial Substitution of NaCl by KCl. J. Agric. Food Chem..

[47] Li X., Peng C., Xiao S., Wang Q., Zhou A. (2024). Two Novel Angiotensin-Converting Enzyme (ACE) Inhibitory and ACE2 Upregulating Peptides from the Hydrolysate of Pumpkin (*Cucurbita moschata*) Seed Meal. J. Agric. Food Chem..

[48] Bhandari D., Rafiq S., Gat Y., Gat Y., Gat P., Waghmare R., Kuma V. (2020). A Review on Bioactive Peptides: Physiological Functions, Bioavailability and Safety. Int. J. Pept. Res. Ther..

[49] Tang S., Chen D., Shen H., Yuan Z., Wei H., Feng Y., Li L., Dong J., Zhang L. (2025). Discovery of two novel ACE inhibitory peptides from soybeans: Stability, molecular interactions, and in vivo antihypertensive effects. Int. J. Biol. Macromol..

[50] Rudolph S., Lunow D., Kaiser S., Henle T. (2017). Identification and quantification of ACE-inhibiting peptides in enzymatic hydrolysates of plant proteins. Food Chem..

[51] Wang F., Zhong H., Cheng J. (2022). Comprehensive Analysis of the Structure and Allergenicity Changes of Seafood Allergens Induced by Non-Thermal Processing: A Review. Molecules.

[52] Fan H., Wang J., Liao W. (2019). Identification and characterization of gastrointestinal-resistant angiotensin-converting enzyme inhibitory peptides from egg white proteins. J. Agric. Food Chem..

